# Use of K_2_CO_3_ to Obtain Products from Starch-Oil Mixtures by Extrusion

**DOI:** 10.3390/foods12203835

**Published:** 2023-10-19

**Authors:** Marzena Włodarczyk-Stasiak

**Affiliations:** Department of Analysis and Evaluation of Food Quality, Faculty of Food Science and Biotechnology, University of Life Sciences in Lublin, Skromna Street 8, 20-704 Lublin, Poland; marzena.stasiak@up.lublin.pl

**Keywords:** starch extrudates, complexation of edible oils, K_2_CO_3_

## Abstract

Mixtures of potato starch with oils (rapeseed and sunflower) were extruded. To improve the complexation of edible oils, a catalyst was added in amounts of 3 g, 6 g, and 9 g per 100 g of sample. The aim was to obtain potato starch extrudates with a high degree of complexation and edible oils during physical modification (extrusion) with the innovative use of K_2_CO_3_ as a catalyst. Selected functional properties (water solubility index and fat absorption index) and technological properties of the obtained extrudates (radial expansion index); color in the L*, a*, and b* systems, and the specific surface area was determined from the water vapor adsorption isotherm (S_BET_). The fat content was determined as external, internal, or bound, and complexed by amylose to assess the degree and manner of fat complexation during extrusion. Iodine-binding capacity and the complexing index were determined to confirm the formation of amylose-lipid complexes. The incorporation of edible oils resulted in a decrease in the radial expansion index and water solubility index compared to control samples. The extrudates were dark orange. Extrudates obtained at the temperature profile L: 80/80/80/60/60/50 °C, depending on the cooking oil, complexed from 48–79% of the introduced rapeseed oil and from 36–40% of the sunflower oil. The extrusion temperature profile (H: 100/100/100/75/75/60 °C) reduced the amount of bound lipid fractions. Using potassium carbonate in the extrusion of starch-lipid systems gives hope for further increasing the share of lipids in extruded mixtures.

## 1. Introduction

Extrusion has been used for many years to obtain increasingly complex systems with unlimited possibilities for creating new properties and structures. This process finds new applications in various industries, from food and chemicals to horticulture. The individual properties of the extruded raw material, parameters (time, temperature, mixing, pressure, humidity, and matrix), and interactions occurring during the process shape the texture and physicochemical properties of the newly created product. Extrusion in the food industry was used mainly for physically modifying raw materials with high starch content as it plays an essential structure-forming role. Due to the need to enrich the composition and give new characteristics to the food, proteins, and fats of various origins were introduced into the mixtures as plasticizers. During extrusion, starch gels and proteins denature, creating an amorphous matrix capable of incorporating lipids [1,2,3,4,5].

An interesting issue from a scientific and research point of view is the method and possibilities of complexing lipids by starches during extrusion. One problem is the limited addition of fats to extruded mixtures. It is assumed that exceeding 3–5% of the addition deteriorates significant qualitative features of the extrudate, and higher additions may result in the formation of compact structures [6,7], reducing the radial expansion and increasing the sample density. Hydrothermal conditions and increased temperature are the basis of extrusion, but they may contribute to auto-oxidation of lipids [1]; moreover, abrasion of the metal working elements of the extruder is possible, which may accelerate oxidation [8,9] as well as the increased surface area of contact with the oxygen of the lipids contained in the extrudate [1].

Another issue is the place and method of “incorporating” lipids in the extrudate. Lipids can be located in the surface areas of the extrudate [10] in the form of separated lipid droplets embedded in matrices [11] and form complex complexes with amylose [7,12]. The formation of such complexes depends on the type of fat, process parameters, and the presence of substances that catalyze or inhibit complexation. The mentioned substances are present because complexes can only be formed with free fatty acids or monoglycerides [13,14].

Due to the problems and limitations of extruding mixtures with a high-fat content in which the matrix is starch, the introduction of a catalyst was proposed. Combining various starch modification techniques to achieve specific properties is becoming increasingly popular. This work combines the physical modification of starch during extrusion with chemical modification by esterifying edible oils with fatty acids. Another novelty of this work is the use of potassium carbonate (K_2_CO_3_) as a catalyst during chemical modification. This substance shows high degrees of substitution (~2.5) and constant reactivity during temperature changes [15,16,17,18]. K_2_CO_3_ is also used as a leavening and moisture-binding substance in food products.

The work aimed to obtain potato starch extrudates with a high degree of complexation with edible oils by combining physical and chemical modification, determining the selected physicochemical properties of products and the nature of starch-fat complexes.

## 2. Materials and Methods

### 2.1. Materials

The material for the tests were extrudates obtained from potato starch produced in Zakłady Przemysłu Ziemniaczanego Lublin and sunflower or rapeseed oil produced in Zakłady Tłuszczowe “Kruszwica”. Mixtures of various qualitative and quantitative compositions were prepared from the above-mentioned raw materials (Table 1). The mixtures were extruded at constant: moisture (22%), screw rotation speed (200 rpm), and nozzle diameter (3.5 mm). When determining the parameters, the names of the low (L) and high (H) temperature ranges in the individual sections of the extruder were conventionally assumed (Table 2).

Extrusion was carried out on a Clextral EV-25 (Evolum, Clextral, Firminy, France). The samples obtained for part of the tests required special preparation, i.e., grinding and sieving through a sieve with a mesh size of 63 mm.

### 2.2. Methods

#### 2.2.1. Radial Expansion Index

The radial expansion ratio (*E_x_*) is the ratio of the extrudate diameter (*D*) to the diameter of the extruder nozzle (*d*). The diameter of the extrudates was measured with a caliper with an accuracy of 0.05 mm in 50 repetitions. The radial expansion index was calculated according to the Formula (1):

(1) $$E_{x}=D/d$$

*E_x_*—radial expansion index
*D*—extrudate diameter (mm)
*d*—extruder nozzle diameter (mm)

#### 2.2.2. Color Measurement

The color was measured using the NH310 Colorimeter (Shenzhen Threenh Technology Co., Ltd., Guangzhou, China) in the L*, a*, and b* system, for whole and fragmented samples, in 10 repetitions. Measurements were performed in three repetitions unless the methodology indicated more repetitions. The results were averaged, and the standard deviation was calculated. The averaged color parameters were used to calculate the total color difference (∆E), (Equation (2)) [19].

(2) $$∆E=\sqrt{\left(\left(∆L^{2}\right)+\left(∆a^{2}\right)+\left(∆b^{2}\right)\right)}$$

∆E 0–1—undetectable differences.
∆E 1–2—minor differences.
∆E 2–3.5—medium, detectable differences.
∆E 3.5–5—distinct differences.
∆E above 5 means significant differences in the hue of the color.

#### 2.2.3. Water Solubility Index and Fat Absorption Index

Determination of the water solubility index, WSI, was performed according to Anderson et al. [20]. A total of 30 mL of water (20 °C) was added to 1 g of the sample, then homogenized (5 min) and centrifuged (20 min, 2000 rpm). The unbound water was poured off, and then the test tube with the material was dried (24 h, 105 °C). The degree of solubility in water was calculated from Formula (3):

(3) $$WSI=\frac{m_{r}}{m}\;100$$

*WSI*—percentage content of entered the solution per 1 g of the material [%, *w*/*w*]
*m_r_*—weight of substance entered into the solution [g]
*m*—weighed portion, converted to value per 1 g of dry matter [g]

Fat absorption index (FAI) was determined according to the method of Beuchat [21]. Samples (5 g) were mixed with oil (25 mL, 5 min) and unbound oil was removed after centrifuging (2000 rpm, 10 min). FAI was calculated from Formula (4):

(4) $$FAI=\frac{m_{m}-m}{m}\;100$$

*FAI*—percentage content of bound oil per 1 g of the material [%, *w*/*w*]
*m_m_*—weight of test tube with wet sediment [g]
*m*—weighed portion, converted to value per 1 g of dry matter [g]

#### 2.2.4. The Specific Surface Area (S_bet_) of the Water Vapor Adsorption Isotherm

Extrudate samples were weighed with an accuracy of 0.0001 g and placed in 5 desiccators with a water activity (a_w_) of 0.004, 0.15, 0.22, 0.30, and 0.35, respectively. Water activity was controlled with sulfuric acid of different densities. The process was carried out at 25 °C to determine the equilibrium humidity. The amount of adsorbed water vapor was calculated from the differences between the final weight of the material and the weight of dried samples per 1 g.

To calculate the specific surface area (S_BET_), adsorption in the range aw 0.01–0.35 was used, which corresponds to monomolecular adsorption according to the BET theory (5) [19,22]:

S_BET_ = (a_m_ × σ_0_ × N_0_) × M^−1^ (5)

S_BET_—specific surface area (m^2^/g)
a_m_—monolayer capacity (gH_2_O/g d.m.)
σ_0_—the surface area of the water molecule (10.8 × 10^−20^ m^2^/molecule)
N_0_—Avogadro’s number (6023∙10^23^)
M—molecular weight of water (18 g/mol)

#### 2.2.5. Fat Content

To assess the degree and manner of fat complexation during extrusion, the fat content was determined:

- external (a′)
- internal (a″)
- bound, complexed by amylose (a‴).

Extraction of individual fat fractions was carried out in a Soxhlet apparatus.

To determine the external fat content (a*), 5 g samples of unground extrudates were extracted by shaking for 2 h in hexane (50 mL) at a speed of 190 rpm and an amplitude of 7. After this time, hexane was regenerated (140 °C); the external fat was determined by weight in the Soxhlet apparatus.

Extrudates devoid of external fat were ground and then extracted on 2 g aliquots, shaking for 2 h in hexane (50 mL) at 190 rpm and amplitude 7. Regeneration of the extraction reagent and determination of the internal fat (a**) amount was carried out as above.

Bound fat (a***), complexed with amylose, was determined after subjecting samples from internal fat determinations to acid hydrolysis. The weight of the tested samples was approx. 4 g. The samples were hydrolyzed using 25 cm^3^ of 1 M hydrochloric acid and 45 cm^3^ of water, for 60 min at 90 °C. The bound fat was extracted with modified parameters (shaking 5 min, amplitude 9.5). The extracts thus obtained were centrifuged (5 min, 1000 rpm) to remove the starch matrix. Hexane was bound with fat, regenerated as above, and the amount of fat was determined by weight with an accuracy of 0.0001 g.

#### 2.2.6. Iodine-Binding Capacity (IBC)

The iodimetric method of amylose content determination of Bhatnagar & Hanna was used [13]. For IBC determination, extrudates were defatted in petroleum ether to remove free lipids.

#### 2.2.7. Complexing Index (CI)

Complexing Index (CI) was determined using the method of Gilbert & Spragg [23]. This method involves the formation of a starch-iodine complex and measurement of starch which is not complexed with lipids. CI was calculated from Equation (6):

(6) $$CI=\frac{A_{0}-A_{s}}{A_{0}}100$$

*CI* = Complexing Index (%)
*A*_0_—absorbance of control
*A_s_*—absorbance of the sample

#### 2.2.8. Iodine Spectra of Starch Samples

The absorbance spectra of starch-iodine complexes were measured using a spectrophotometer from 400–700 nm, and the wavelengths of maximum absorption (Amax) values were determined. The ratios of 630 nm and 520 nm absorbances for amylose and amylopectin were calculated [13,24].

#### 2.2.9. Statistical Analysis

In the study, the mean values (x) and the standard deviation (σ) were calculated from the range (x − 2σ; x + 2σ); data out of the range were rejected. The ANOVA was used to calculate significant differences in treatment means and LSD (α < 0.05). The data reported in all the results are an average of triplicate observations.

## 3. Results and Discussion

Extrusion was carried out at different temperature profiles and amounts of added edible oil (sunflower or rapeseed). The obtained samples had different levels and types of added oil compared to potato starch (Table 1 and Table 2). To facilitate oil complexation (with its relatively high share for extrusion, up to 9%) and to obtain the desired quality characteristics of the extrudates (high EX value), a substance conventionally referred to as the K_2_CO_3_ catalyst was introduced into the mixture.

The obtained extrudates were characterized by low radial expansion values, ranging from 1.76 to 2.37 (Table 3). Regardless of the type and amount of added oil and the temperature in individual sections of the extruder (L: 80/80/80/60/60/50 °C; H: 100/100/100/75/75/60 °C), a slight decrease in EX was observed for all samples when compared to the control cH and cL (without the addition of oil). Mild process conditions and temperature profile L did not significantly affect the EX value. Increasing the temperature in the extruder sections (profile H) significantly decreased EX (α = 0.05) for samples with rapeseed oil. With the increase in the share of sunflower oil, a decrease of EX: S1H > S2H > S3H was recorded. Many researchers indicate that too much fat may reduce the degree of product expansion. Even 3% oil addition causes the formation of compact structures in which no air chambers are observed [6,7]. Such a negative effect of fat on radial expansion can be explained as “flooding” and “loading” the air chambers, the formation of which is mainly responsible for starch. The “lubricating” role of oils is also probable, which may cause slippage of the extruded mass, worse mixing, and “slowing down” when extruding through the nozzle. The result is a relative pressure drop and a weaker expansion effect after passing through the nozzles, which is crucial for the radial expansion value.

Amft (2019) [25] examined the properties of meal corn extrudates with various additions of commercial sunflower and rapeseed oils, pointing out the critical role of the humidity level on the radial expansion. The higher the humidity during the process, the smaller the expansion and the smoother the surface. 

The chromatic coordinates (L*, a*, and b*) of the different extrudates under study are presented in Table 4. Regardless of the amount of oil added to the extruded mixture, its products were significantly (α = 0.05) darker than the control samples, as evidenced by a decrease in the L parameter. There was also an increase in the red (+a) value from the yellow (+b) color vector. A three-factor color analysis allows us to conclude that the extrudates became darker as the fat fraction increased (dark orange). 

The color appearing in extrudates is the formation of colored melanoid compounds. These compounds are considered to be antioxidants. This is very desirable because it extends the stability of extruded products during long-term storage and enriches them with these compounds [7,26,27].

Table 5 presents the samples’ selected functional properties, such as solubility (WSI, %) and fat absorption capacity (FAI, %). From a technological point of view, WSI contains important information about the possibility of creating homogeneous solutions in aqueous food systems. The FAI index, defined as the ability to bind fat and retain it against the forces of gravity, has an entirely different meaning. Regardless of the type and amount of added edible oil, very low WSI values (%) were recorded for all samples and were almost three times lower than the control samples. Statistically significant differences (α = 0.05) were noted for the WSI of control samples (cL and cH) about extrudates with the addition of sunflower and rapeseed oils. The average WSI values ranged from 5–9% to nearly 20% (cL and cH). For samples S3H with 9% sunflower oil and S1H with 3%, the lowest WSI values were recorded 5.00% and 6.05%, respectively.

Similar results were also obtained by other researchers [6,13,28,29]. Such low WSI values mean that the very compact and coherent structure formed during the complexation of starch with acids fatty may makes it difficult for water molecules to penetrate the newly formed extrudate structure, thus weakening its dissolution. Significantly higher WSI values for control samples can be explained as obtaining a larger hydrodynamic volume, which could facilitate the destruction of starch granules, thus increasing the share of areas susceptible to solubility [28].

Most food products are exposed to moisture in the air. This adversely affects the quality and safety of food, causing caking and softening, and the increase in water activity contributes to the multiplication of undesirable microflora and intensification of biochemical processes. Given these facts, knowledge of the specific surface area (S_BET_), i.e., the relative surface of the moisture interaction with the product, is a source of information on how and on what scale it is necessary to prevent these processes [22,30,31]. Table 6 presents the values of the analyzed samples’ specific surface area (S_BET_). It is quite a surprise to obtain slightly different values of specific surface area, both in the context of the control test and the test with the addition of another oil: temperature levels during extrusion (L: 80/80/80/60/60/50 °C; H: 100/100/100/75/75/60 °C) and the addition of sunflower or rapeseed oil (3%, 6%, 9%). According to Bhatnagar & Hanna [32], they indicate that even a 4% addition of fat fractions reduces the porosity of extrudates while increasing their density. Dextrumaux et al. [33] note that the extrudates’ average “cell” size decreased as the fatty acid content increased. The results of the mentioned works indicate that adding fat fractions weakens or limits the availability of adsorption centers for water vapor particles. They do not indicate a weakening of the absorption of water vapor molecules because the SBET values for the control and proper samples do not differ statistically significantly (α = 0.05) (Table 6). In this work, different results from our own research were obtained. They do not indicate a weakening of the absorption of water vapor molecules because the SBET values for the control and proper samples do not differ significantly statistically (α = 0.05) (Table 6). Most likely, part of the catalyst added to the extruded mixtures (K_2_CO_3_) decomposes quite easily at elevated temperatures and acidic environments to, among others, CO_2_. Carbon dioxide released from the hot mass leaving the extruder gives it a porous structure, which becomes fixed during sudden cooling in the nozzle.

The available literature data indicate great difficulties during extrusion and low quality of products even with 4–5% lipid addition. Potassium carbonate (K_2_CO_3_) as a catalyst is used to obtain higher fatty esters with a high degree of substitution, DS > 2.5 [18]. This catalyst was used with great success by Filip et al. [17] and Junistiaa et al. [16] for obtaining higher fatty acid esters, and Wlodarczyk-Stasiak et al. [15] during the extrusion of potato starch with food oils (rapeseed and linseed) and glycerol.

To learn how starch and lipids are complexed in the extrudate, we added K_2_CO_3_ that was marked as external, internal, or complexed by amylose (Table 7). It was also assumed that the hexane used for subsequent extractions cannot break ionic, hydrogen, or hydrophobic interactions and only free lipids [34,35].

Regardless of the type of added oil, the amount added to the mixture, and extrusion conditions, the external fat content was marginal for most samples and did not exceed 1% of the added amount. Such a small amount (%) of fat fractions contributes to the more effective resistance of extrudates to external factors such as light, humidity and temperature, as well as to achieving a higher level of permanent fat complexation inside. This assumption, which is not analyzed in this work, is confirmed by research [25]. Researchers extruded meal corn with edible oils (rapeseed and sunflower) at various levels of process humidity. It turns out that at the same humidity level of 22% as in this work, their samples after 30 days of storage are less oxidized (~58 mmol O_2_/kg fat) than when the humidity during extrusion was 10% (80 mmol O_2_/kg fat). Researchers explain this through smoothing of the sample surfaces (low EX), the starch matrix’s protective function reducing oxygen availability, and the strong incorporation of the lipid fraction in the surface areas of the extrudate, which reduces reactivity with oxygen molecules. This protective function of the starch matrix for lipids located in the superficial areas of extrudates has been confirmed [7,36,37].

Internal fat has a much higher share in terms of the amount added to the extruded mixture (%). Extrudates with the addition of rapeseed oil, obtained with temperature profile H: 100/100/100/75/75/60 °C, show a similar percentage of bound oil of 23–28%. The temperature profiles (H) obtained at the same time for extrudates with sunflower oil show a much lower percentage of bound oil, S1H-7.8% and S3H-9.51%. The temperature profile L: 80/80/80/60/60/50 °C during extrusion and the addition of rapeseed oil decreased the bound oil percentage as its share in the mixture increased. This tendency of a decrease in the % share of fat with an increase in its share in the mixture was observed for bound complexed fat by amylose (S1H > S2H > S3H; S1L > S2L > S3L). Analyzing the total percentage of fat binding to the amount entered during the extrusion, it is clear that mild extrusion conditions (L: 80/80/80/60/60/50 °C) contribute to higher bound fat. Samples with a three percent oil addition had the highest amount of complexed fat relative to the amount added (R1L-79.14%; S1L-54.12%).

A small amount of lipids on the surface of extrudates and their strong complexation inside are mentioned by Amft, 2019. They suggest that lipids are embedded in the matrix, located between the amylose helix or dispersed in amorphous areas, and do not form helical complexes with amylose. However, Thachil et al. [7] put forward the thesis that there are different locations of fat in the starch matrix in extruded products, in the complexation of which amylose takes part. Other studies confirmed that the lipid fractions of extrudates constitute amylose-lipid complexes but only with free fatty acids and monoglycerides [13,28,29,38,39]. 

The results of subsequent tests confirm the thesis about the strong complexation of vegetable oils by starches during extrusion (Table 8). There was a nearly 15-fold decrease in the amount of IBC, regardless of the amount and type of vegetable oil, compared to the reference samples (cH; cL). Such changes in IBC are described by Bhatnagar & Hanna [13,40]. The researchers assume that any free fat present in the samples was removed and that the reduction in IBC was due to the inaccessibility of the iodine binding site due to lipid complexation during extrusion. Extrudates obtained with the addition of rapeseed oil are characterized by a much higher level of complexation (min 90.31%; max 96.43%) than sunflower oil (min 74.59%; max 92.33%). It was observed that for extrudates with the addition of sunflower oil, the complex starch (S1H > S2H > S3H; S1L > S2L > S3L) decreased linearly (R2 ~ 1) as the oil content increased. It is probable that sunflower oil, penetrating the starch helices, competes for the active sorption centers located in its outer areas, which weakens iodine complexation.

Another reason for such a strong, complex starch can be found in the high water content during extrusion. This makes it easier for the starch granules to swell more completely, increasing the availability of amylose and binding lipids between the helices. The water level also influences the course of gelatinization. Water contributes to the disorganization of the structure, facilitating amylopectin from it and increasing the availability of amylose responsible for the incorporation of lipids between its helices [41].

The absorption spectra (I_2_) of extrudates were also determined to confirm the formation of complexes (Table 8). The absorbance ratio at 630 nm and 520 nm was determined for max A for amylose and amylopectin, respectively. The 630/520 ratios for all non-defatted samples were slightly higher than for their hexane-extracted counterparts, which indicates a higher iodine-binding capacity. Minor differences in the 630/520 ratio confirm that the amylose-lipid complex constitutes a small share of the total amount of Bound complexed fat by amylose (Table 7). According to Sokhey & Chinnaswamy [42], changes in the discussed coefficient indicate amylose and amylopectin; they show a difference in the composition of the linear or branched fractions of starch molecules.

Slight shifts of max A were also observed, from 630 nm for amylose towards lower wavelengths (nm). The most significant changes for A max were observed for samples extruded in the L temperature profile and with 9% (S3L-A max-618 nm) and 6% (S2L-A max-619 nm; R2L-A max-618 nm) addition of edible oils. According to Bhatnagar & Hanna [40], iodine spectra and absorbance ratio at 630 nm and 520 nm can be used to detect the formation of complexes during extrusion.

## 4. Conclusions

The addition of edible oils resulted in a decrease in EX and WSI compared to control samples. The extrudates were dark orange, indicating the formation of melanoid compounds. Using a catalyst resulted in the lipid fractions on the surface of the extrudates constituting less than 1% of their total amount. The 3% addition of K_2_CO_3_ contributed to obtaining extrudates with high complexation of rapeseed oil from 48–79% of the introduced amount and 36–40% of sunflower oil, with a temperature profile of L: 80/80/80/60/60/50 °C. However, carrying out extrusion at profile H: 100/100/100/75/75/60 °C resulted in an approximately 10% reduction in bound lipid fractions for samples with rapeseed oil and about 5% for sunflower oil. For sunflower oil, regardless of the temperature profile during extrusion, there was a stronger tendency to form complexes with amylose than rapeseed oil, the higher values of which (%) were recorded as internal fat. The use of potassium carbonate in the extrusion of starch-lipid mixtures makes it possible to increase the amount of complexed oil, and the plasticizing role of fat fractions is applicable in the production of edible food films.

## Figures and Tables

**Table 1:** The qualitative and quantitative characteristics of starch-oil blends used for extrusion (%).

Code of Sample	Potato Starch	Catalyst K_2_CO_3_	Oils	
			Rapeseed Oil	Sunflower Oil
(g/100 g)				
Control (c)	100	0	0	0
R1	96	3	3	-
R2	91	3	6	-
R3	88	3	9	-
S1	96	3	-	3
S2	91	3	-	6
S3	88	3	-	9

R—sample with the addition of rapeseed oil, S—sample with the addition of sunflower oil.

**Table 2:** Characteristics of conditions of extrusion.

Code of Sample	Temperature Profile (°C)	Screw Speed (rpm/min)	Nozzle Diameter (mm)	Moisture (%)
H	100/100/100/75/75/60	200	3.5	22
L	80/80/80/60/60/50			

H—an abbreviation for a conventionally defined “higher” temperature profile in the extruder sections, L—an abbreviation for a conventionally defined “lower” temperature profile in the extruder sections. Extruder type Clextral EV-25 (Evolum), output 7 kg/h.

**Table 3:** Radial expansion (EX) of extrudates.

Radial Expansion (EX)			
cH	2.84 ± 0.02 ^b^	cL	2.97 ± 0.04 ^b^
R1H	1.88 ± 0.01 ^a^	R1L	2.03 ± 0.04 ^ab^
R2H	1.76 ± 0.04 ^a^	R2L	2.03 ± 0.03 ^ab^
R3H	1.82 ± 0.05 ^a^	R3L	2.03 ± 0.02 ^ab^
S1H	2.35 ± 0.11 ^b^	S1L	2.13 ± 0.04 ^ab^
S2H	2.03 ± 0.04 ^ab^	S2L	2.01 ± 0.01 ^ab^
S3H	1.82 ± 0.03 ^a^	S3L	2.04 ± 0.02 ^ab^

The same letters in columns indicate values that are not significantly different at α = 0.05.

**Table 4:** Color measurement in the L*, a*, and b* system of extrudates.

Color Measurement in the L*, a*, b* System									
Sample	L	a	b	∆E	Sample	L	a	b	∆E
cH	46.66 ± 0.42 ^b^	0.33 ± 0.07 ^a^	2.28 ± 0.04 ^a^	-	cL	48.81 ± 1.28 ^b^	0.18 ± 0.07 ^a^	2.55 ± 0.21 ^a^	-
R1H	42.02 ± 0.59 ^a^	1.88 ± 0.11 ^a^	5.64 ± 0.15 ^a^	5.94	R1L	41.89 ± 0.23 ^a^	1.66 ± 0.10 ^a^	5.30 ± 0.41 ^a^	7.59
R2H	42.15 ± 0.64 ^a^	2.44 ± 0.22 ^ab^	6.14 ± 0.33 ^ab^	6.31	R2L	43.35 ± 0.63 ^a^	1.81 ± 0.13 ^ab^	5.35 ± 0.28 ^a^	6.35
R3H	42.94 ± 0.46 ^a^	1.83 ± 0.24 ^a^	3.63 ± 0.38 ^a^	4.24	R3L	45.04 ± 0.52 ^ab^	2.51 ± 0.15 ^b^	7.74 ± 0.32 ^b^	6.82
S1H	42.12 ± 0.53 ^a^	1.51 ± 0.10 ^a^	4.86 ± 0.16 ^a^	5.36	S1L	43.68 ± 0.30 ^a^	1.52 ± 0.10 ^a^	6.19 ± 0.20 ^ab^	6.44
S2H	44.38 ± 0.74 ^a^	2.54 ± 0.32 ^ab^	7.65 ± 0.39 ^ab^	6.26	S2L	43.95 ± 1.14 ^a^	1.63 ± 0.14 ^a^	6.63 ± 0.34 ^ab^	6.51
S3H	44.76 ± 0.21 ^a^	3.64 ± 0.09 ^b^	8.91 ± 0.15 ^b^	7.67	S3L	45.45 ± 0.70 ^ab^	2.85 ± 0.15 ^b^	4.94 ± 1.32 ^a^	4.91

The same letters in columns indicate values that are not significantly different at α = 0.05.

**Table 5:** Selected functional properties: WSI and FAI.

Selected Functional Properties					
Samples	WSI	FAI	Samples	WSI	FAI
cH	19.09 ± 2.10 ^b^	165.25 ± 7.53 ^ab^	cL	20.09 ± 3.09 ^b^	177.75 ± 1.76 ^b^
R1H	8.24 ± 0.41 ^a^	156.69 ± 2.54 ^a^	R1L	9.98 ± 0.25 ^a^	169.258 ± 3.39 ^a^
R2H	8.58 ± 0.63 ^a^	157.27 ± 0.56 ^a^	R2L	9.47 ± 0.92 ^a^	165.82 ± 4.24 a
R3H	7.97 ± 0.81 ^a^	163.54 ± 2.72 ^ab^	R3L	8.61 ± 1.13 ^a^	164.57 ± 7.74 ^a^
S1H	6.05 ± 0.27 ^a^	167.28 ± 5.37 ^b^	S1L	9.21 ± 0.34 ^a^	162.11 ± 1.69 ^a^
S2H	7.08 ± 6.95 ^a^	161.51 ± 7.07 ^ab^	S2L	9.05 ± 7.49 ^a^	167.81 ± 3.11 ^a^
S3H	5.00 ± 0.11 ^a^	165.41 ± 5.83 ^ab^	S3L	9.06 ± 7.01 ^a^	161.49 ± 6.46 ^a^

WSI—water solubility index (%): FAI—fat adsorption index (%). The same letters in columns indicate values that are not significantly different at α = 0.05.

**Table 6:** Specific surface area (S_BET_) of extrudates.

Specific Surface Area (S_BET_; m^2^/g)			
cH	210.04 ± 8.58 ^ab^	cL	179.85 ± 8.25 ^a^
R1H	199.45 ± 10.77 ^a^	R1L	234.73 ± 9.67 ^b^
R2H	204.09 ± 8.18 ^ab^	R2L	181.49 ± 8.67 ^a^
R3H	199.30 ± 9.05 ^a^	R3L	186.90 ± 7.09 ^a^
S1H	204.16 ± 11.25 ^ab^	S1L	187.83 ± 11.34 ^ab^
S2H	197.14 ± 10.15 ^a^	S2L	184.18 ± 10.59 ^a^
S3H	205.35 ± 6.29 ^ab^	S3L	203.38 ± 11.05 ^ab^

The same letters in columns indicate values that are not significantly different at α = 0.05.

**Table 7:** The fat content of extrudates.

Sample	Fat Content (g/100 g)			The Percentage of Fat Binding (%) *			The Total
	External Fat Content	Internal Fat Content	Bound Complexed Fat by Amylose	The External	The Internal	The Complexed	
cH	0.000 ± 0.000 ^a^	0.000 ± 0.000 ^a^	0.000 ± 0.000 ^a^	-	-	-	-
R1H	0.006 ± 0.001 ^a^	0.713 ± 0.003 ^a^	0.560 ± 0.002 ^a^	0.20	23.77	18.67	42.63
R2H	0.084 ± 0.003 ^ab^	1.736 ± 0.008 ^ab^	0.903 ± 0.004 ^ab^	1.40	28.93	15.05	45.38
R3H	0.015 ± 0.002 ^a^	2.387 ± 0.007 ^b^	1.357 ± 0.006 ^b^	0.17	26.52	15.08	41.77
S1H	0.018 ± 0.001 ^a^	0.234 ± 0.004 ^a^	0.900 ± 0.005 ^ab^	0.60	7.80	30.00	38.42
S2H	0.012 ± 0.001 ^a^	1.271 ± 0.009 ^ab^	1.063 ± 0.006 ^ab^	0.20	21.18	17.72	39.08
S3H	0.118 ± 0.005 ^b^	0.856 ± 0.005 ^a^	1.428 ± 0.007 ^b^	1.31	9.51	15.87	26.68
cL	0.000 ± 0.000 ^a^	0.000 ± 0.000 ^a^	0.000 ± 0.000 ^a^	-	-	-	-
R1L	0.017 ± 0.002 ^a^	1.559 ± 0.005 ^a^	0.798 ± 0.005 ^a^	0.57	51.97	26.60	79.14
R2L	0.020 ± 0.001 ^a^	2.064 ± 0.008 ^b^	1.165 ± 0.008 ^ab^	0.33	34.40	19.42	54.16
R3L	0.007 ± 0.001 ^a^	2.403 ± 0.005 ^b^	1.990 ± 0.006 ^b^	0.08	26.70	22.11	48.89
S1L	0.084 ± 0.003 ^b^	0.582 ± 0.007 ^a^	0.957 ± 0.008 ^ab^	2.80	19.40	31.90	54.12
S2L	0.023 ± 0.002 ^a^	0.886 ± 0.007 ^a^	1.295 ± 0.010 ^ab^	0.38	14.77	21.58	36.74
S3L	0.047 ± 0.002 ^ab^	1.865 ± 0.010 ^ab^	1.745 ± 0.005 ^b^	0.52	20.72	19.39	40.63

* The percentage of fat binding to the amount entered during the extrusion. The same letters in columns indicate values that are not significantly different at α = 0.05.

**Table 8:** IBC and iodine absorption spectra of extrudates.

Sample	IBC (mg/100 mg)	Complexed Starch Index (%)	Iodine Absorption Spectra (nm)			
			After Defatted		Before Defatted	
			Ratio 630/520	A Max	Ratio 630/520	A Max
cH	1.96	-	1.98	628	1.95	629
R1H	0.10	94.66	1.91	629	1.88	625
R2H	0.12	93.85	1.92	625	1.90	625
R3H	0.07	96.43	1.95	627	1.94	623
S1H	0.25	87.45	1.92	626	1.87	625
S2H	0.32	83.41	1.91	626	1.84	625
S3H	0.42	78.33	1.86	622	1.80	623
cL	1.83	-	1.76	625	1.99	635
R1L	0.18	90.31	1.87	626	1.80	623
R2L	0.13	92.91	1.89	626	1.68	618
R3L	0.20	89.34	1.91	627	1.85	625
S1L	0.14	92.33	1.95	627	1.84	622
S2L	0.33	82.27	1.92	626	1.80	619
S3L	0.47	74.59	1.90	627	1.66	618

## Data Availability

The data used to support the findings of this study can be made available by the corresponding author upon request.
